# Association of tumor Schwann cells with epithelial-mesenchymal transition and immune-suppressive microenvironment in triple-negative breast cancer

**DOI:** 10.1007/s10549-026-08050-z

**Published:** 2026-08-10

**Authors:** Kei Kawashima, Eslam A. Elsaedy, Masanori Oshi, Akimitsu Yamada, Kazutaka Narui, Shipara Gandhi, Elizabeth Repasky, Takashi Ishikawa, Itaru Endo, Kazuaki Takabe

**Affiliations:** 1 Department of Surgical Oncology, Roswell Park Comprehensive Cancer Center, Buffalo, NY 14263 USA; 2 Department of Gastroenterological Surgery, Yokohama City University Graduate School of Medicine, Yokohama, Kanagawa Japan; 3 Department of Breast and Endocrine Surgery, Yokohama City University Graduate School of Medicine, Yokohama, Kanagawa Japan; 4 Department of Breast and Thyroid Surgery, Yokohama City University Medical Center, Yokohama, Kanagawa Japan; 5 Department of Medicine, Winship Cancer Institute of Emory University, Atlanta, GA USA; 6 Department of Immunology, Roswell Park Comprehensive Cancer Center, Buffalo, NY USA; 7 Department of Breast Surgery and Oncology, Tokyo Medical University, Tokyo, Japan; 8 Department of Surgery, University at Buffalo Jacobs School of Medicine and Biomedical Sciences, The State of University New York, Buffalo, NY USA; 9 Division of Digestive and General Surgery, Niigata University Graduate School of Medical and Dental Sciences, Niigata, Japan; 10 Department of Breast Surgery, Fukushima Medical University, Fukushima, Japan; 11 Department of Surgical Oncology, Roswell Park Comprehensive Cancer Center Elm & Carlton Streets, Buffalo, NY 14263 USA

**Keywords:** Triple-negative breast cancer, Intratumoral nerve, Schwann cell, Tumor microenvironment, Transcriptome

## Abstract

**Purpose:**

Tumor-associated nerves have recently emerged as an understudied key regulator of cancer biology. However, its quantification using histological or transcriptomic approaches is challenging because their small diameters hinder reliable detection and their cell bodies that hold most mRNA reside outside the tumor. This study evaluated a Schwann cell (SC)-related transcriptomic score as a surrogate for tumor-associated nerves in tumors from triple-negative breast cancer (TNBC) patients.

**Methods:**

Transcriptomic and clinical data from three independent TNBC cohorts, TCGA (*n* = 170), METABRIC (*n* = 335), and SCAN-B (*n* = 174) were analyzed. An SC score was calculated from SC-related gene signatures, and spatial transcriptomics was used to validate SC localization. In addition, seven independent neoadjuvant chemotherapy (NAC) cohorts were analyzed to evaluate the association between SC score and treatment response. Associations between the SC score and clinical features, genomic features, proliferation, treatment response, and the tumor microenvironment (TME) were evaluated.

**Results:**

SC signature genes spatially localized to intratumoral nerve regions, confirming that the SC score reflects their presence within tumors. SC-high tumors were associated with lower mutation burden and with less cell proliferation in the TCGA, METABRIC, and SCAN-B cohorts. SC-high tumors were also associated with low immune activity and fewer infiltrating immune cells, along with enrichment of epithelial–mesenchymal transition (EMT), TGF-beta signaling and stromal remodeling pathways.

**Conclusions:**

The SC score-high TNBC is associated with lower cell proliferation, enhanced stromal remodeling and EMT and suppressed immune activity, suggesting a role of neural-associated TME features in shaping TNBC biology.

**Supplementary Information:**

The online version contains supplementary material available at 10.1007/s10549-026-08050-z.

## Introduction

Peripheral nerve fibers in solid tumors have recently emerged as a previously overlooked key player that affects cancer biology [1]. In addition to pre-existing nerve fibers present in the organ of location, cancer cells can actively induce axonogenesis, a process that promotes the in-growth of new nerve fibers into the tumor microenvironment (TME) [2, 3]. These tumor-associated nerves regulate multiple cancer hallmarks, including immune suppression, cancer cell proliferation, and angiogenesis within the TME, by releasing neurotransmitters and neuropeptides [1, 4]. Higher densities of intratumoral nerve fiber and/or increased neural activity has been reported to correlate with increased tumor aggressiveness and poorer clinical outcomes in multiple cancer types, including pancreatic cancer [5, 6], prostate cancer [7, 8], head and neck squamous cell carcinoma [9, 10], colorectal cancer [11, 12], and breast cancer [13, 14]. In breast cancer, we recently reported that the tumor-induced neuronal projection process is associated with an immune-cold TME and worse clinical outcomes [15].

Despite their importance, intratumoral nerves remain challenging to quantify by routine histological analyses [16]. Nerve fiber diameters can be as small as 0.5–1 μm, making them difficult to detect morphologically with immunohistochemistry [17], and their elongated, filamentous structure undulating through various planes of tissue further complicates accurate quantification of their area or volume from tissue Sect [18]. Furthermore, intratumoral nerve fibers occupy only a very small fraction of the tumor area, estimated at approximately 2% [19], which limits their detectability and complicates efforts to characterize their functional roles within the TME. Transcriptomic analysis of a bulk tumor can overcome the limitation of 2-dimension histological analyses, but has its own fundamental challenges. Most nerve fibers present within solid tumors are predominantly sensory or autonomic nerve endings [20], whose cell bodies reside in dorsal root ganglia or sympathetic ganglia rather than within the bulk tumor mass [21, 22]. Therefore, even when nerve fibers are present within the tumor, transcriptional activity of the nerve fibers is barely detectable because majority of it occurs at some distance from the tumor. Although a subset of mRNA is transported along axons to distal nerve terminals [23], its abundance is limited, representing only 1.3–1.5% of that in the neuronal cell body [24]. As a result, RNA sequencing is unlikely to fully capture nerve fiber–derived transcriptional signals within tumors due to the extremely limited mRNA within nerve fibers, even with single cell RNA sequencing technique [25].

However, peripheral nerve fibers do not exist in isolation but are structurally and functionally supported by Schwann cells (SCs), the most abundant cell type in the peripheral nervous system, ensheathing axons in both myelinated and unmyelinated fibers [26]. In myelinated fibers, SCs form multilayered myelin sheaths whereas in unmyelinated fibers such as C fibers of the sensory nerve, they envelope axons without forming myelin, thereby maintaining their structural organization and integrity [3]. Consequently, SCs are consistently associated with peripheral nerve fibers and are present along their course within tumors, making their transcriptomic signals readily detectable in the bulk tumor. A recent study in head and neck squamous cell carcinoma demonstrated that SC-related transcriptional programs correlate with intratumoral nerve density, supporting their use as a surrogate for quantifying tumor-associated nerves [27].

In breast cancer, histologically identifiable intratumoral nerves has been reported in 30–80% of tumors [14, 28] and appear to be particularly prevalent in triple-negative breast cancer (TNBC) [19, 29], where they have been implicated in multiple aspects of cancer progression, including suppression of antitumor immunity [30], promotion of metastasis [29], and poorer clinical outcomes [19]. However, much of the current evidence is derived from experimental or pre-clinical models, and large-scale transcriptomic or clinical studies in human tumors remain quite limited. As a result, although neural components have begun to attract increasing attention in breast cancer research, the role of nerves within the TME is still not completely understood and their transcriptomic representation within tumors remains poorly defined. To overcome the challenges in quantifying nerve fibers histologically, we leveraged Schwann cell–associated gene signature score as a surrogate to estimate the tumor-associated nerves in the TME and studied its clinical relevance in TNBC. Using this approach, we investigated whether neural-associated transcriptomic features define a biologically distinct phenotype in TNBC and evaluated their clinical and tumor microenvironmental relevance.

## Results

### Schwann cell (SC) score is associated with SC marker gene expressions

**Fig. 1:**
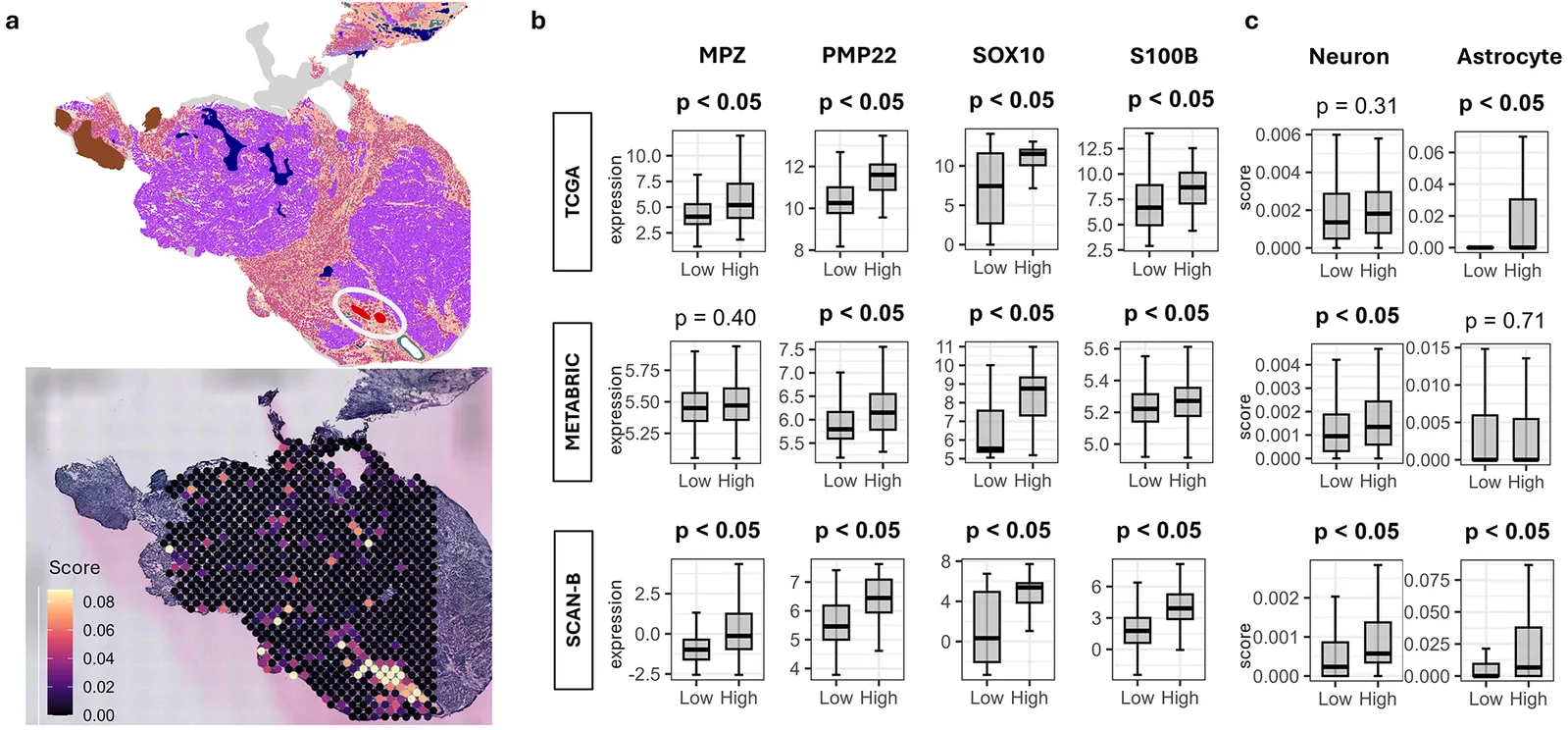
Validation of the Schwann cell (SC) signature in spatial and bulk transcriptomic datasets. (**A**) Upper panel shows histological annotations from the original spatial transcriptomic dataset. Intratumoral nerve regions are highlighted with white circles. Lower panel shows the spatial distribution of the Schwann cell signature genes. The Sc module score was calculated using the AddModuleScore function in Seurat based on the SC signature genes and overlaid on the corresponding hematoxylin and eosin (H&E) image. (**B**) Box plots of SC-high versus SC-low TNBC patients in TCGA, METABRIC and SCAN-B cohorts showing SC-related gene expression. (**C**) Box plots of SC-high versus SC-low TNBC patients in TCGA, METABRIC and SCAN-B cohorts showing xCell neuron and astrocyte score. In the boxplots, the center line indicates the median, the box represents the interquartile range (IQR), and whiskers extend to 1.5×IQR. Outliers were omitted. P values were calculated using the Wilcoxon rank-sum test. Abbreviations: MPZ, myelin protein zero; PMP22, peripheral myelin protein 22; SOX10, SRY-box transcription factor 10; S100B, S100 calcium-binding protein B

First, we investigated whether the SC signature localize with nerves and is associated with well-established SC markers in TNBC. Using a spatial transcriptomic cohort in which intratumoral nerves had been morphologically annotated, we visualized the expression of SC signature genes across spatial spots (Fig. 1a). Spots with high SC score spatially coincided with morphologically annotated intratumoral nerves. This observation aligns with the notion that the SC score reflects SCs localized around nerve fibers. To further validate the SC score in transcriptome of bulk tumors, we examined its association with SC marker genes (Fig. 1b). In the TCGA, METABRIC, and SCAN-B, SC-high tumors were associated with the expression of well-established SC marker genes, including MPZ (myelin protein zero) and PMP22 (peripheral myelin protein 22), which are key components of peripheral myelin, as well as SOX10 (SRY-box transcription factor 10), a transcription factor critical for SC lineage specification, and S100B (S100 calcium-binding protein B), a calcium-binding protein expressed in glial cells including SCs. Also, SC score was correlated with neuron score (Fig. 1c; TCGA: *p* = 0.31, METABRIC: *p* < 0.05, SCAN-B: *p* < 0.05) and astrocyte score (TCGA: *p* < 0.05, METABRIC: *p* = 0.71, SCAN-B: *p* < 0.05) defined in xCell algorithm. These results support the notion that the SC score reflects the presence of SCs while also indicating an association with broader nerve structures within tumors.

### SC score was not associated with survival outcomes across cohorts

**Fig. 2:**
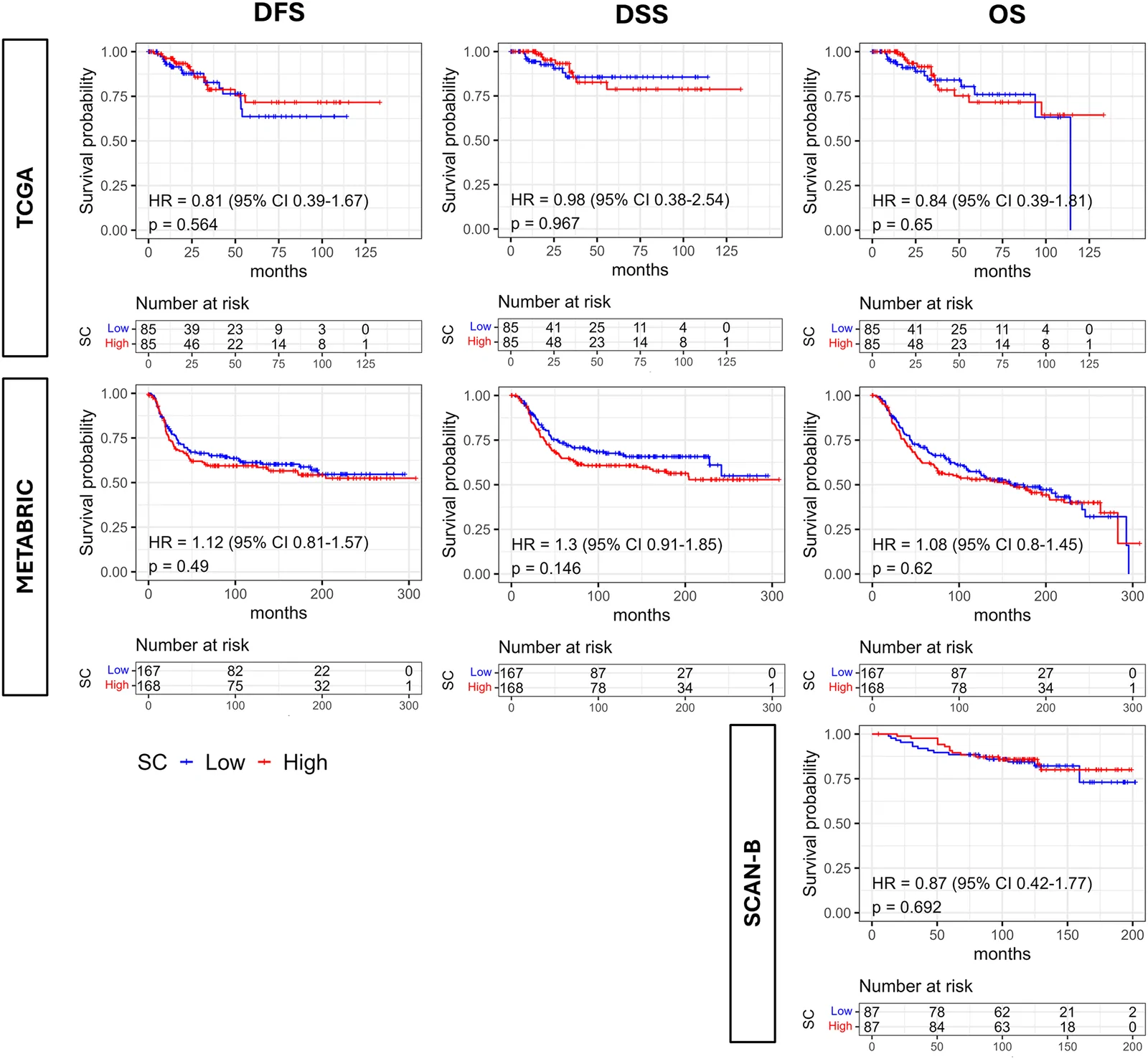
SC score and prognostic outcomes in TCGA, METABRIC, and SCAN-B cohorts. Kaplan-Meier survival curves of overall survival (OS), disease-specific survival (DSS) and disease-free survival (DFS) by SC score group. Red represents SC-high and blue represents SC-low group

Given the previous reports that nerve density in the tumor correlated with poor prognosis in multiple cancer types, we investigated the relationship between the SC score and patient survival in TNBC (Fig. 2). Across all cohorts, no significant association was observed between the SC score and survival outcomes, including disease-free survival (DFS), disease-specific survival (DSS), and overall survival (OS).

### SC score was not associated with driver mutations or genomic instability but with lower mutation burden

**Fig. 3:**
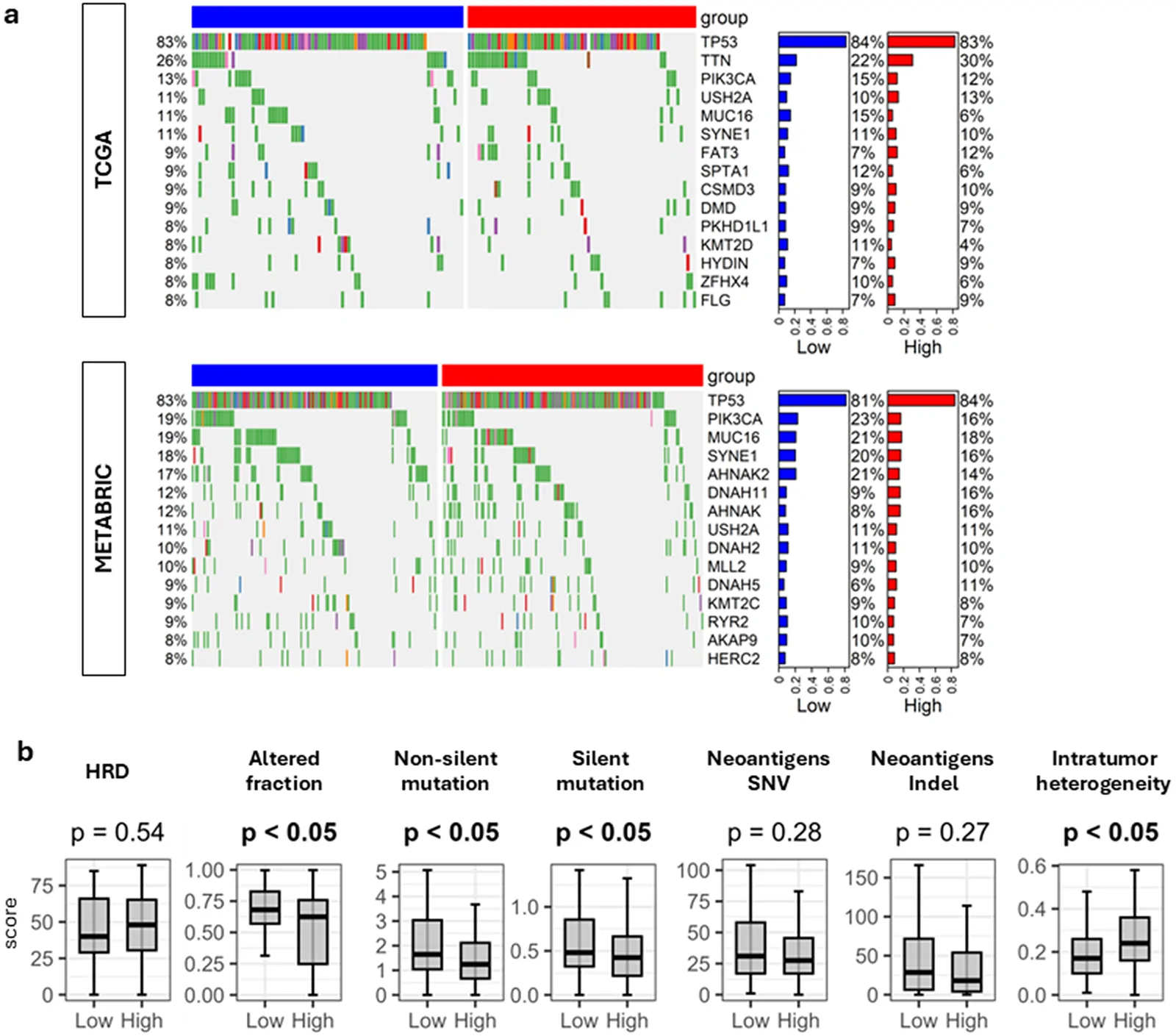
Association of the SC score with genomic alterations. (**A**) Oncoplot showing the mutation landscape of frequently mutated genes in SC score–low and SC score–high tumors in the TCGA and METABRIC cohorts. The top bar indicates the mutation burden for each tumor, and the side bar shows the mutation frequency of each gene within each group. Colors represent different types of somatic alterations as indicated in the legend. Blue indicates SC score–low tumors and red indicates SC score–high tumors. (**B**) Box plots comparing genomic instability–related features between SC score–low and SC score–high tumors in the TCGA cohort, including homologous recombination deficiency (HRD) score, altered fraction, number of non-silent mutations, number of silent mutations, predicted neoantigens derived from single nucleotide variants (SNVs) and insertions/deletions (indels), and intratumor heterogeneity. In the boxplots, the center line indicates the median, the box represents the interquartile range (IQR), and whiskers extend to 1.5×IQR. Outliers were omitted. P values were calculated using the Wilcoxon rank-sum test

Given the lack of association between the SC score and survival outcomes, we next investigated whether differences in genomic alterations, including driver mutations, could underlie the tumor phenotypes associated with the SC score. The frequencies of individual gene mutations showed no consistent differences between the SC-high and SC-low groups in either the TCGA or METABRIC. Oncoplot visualization further demonstrated that the overall mutation landscape, including commonly mutated genes such as TP53 and PIK3CA, was largely comparable between the two groups (Fig. 3a). Taken together, these findings suggest that the SC score is not uniformly associated with genomic instability or recurrent driver gene mutations.

We next examined broader genomic instability–related features in the TCGA (Fig. 3b). Homologous recombination deficiency (HRD) scores did not differ by SC score, whereas the fraction of genome altered was significantly lower in the SC-high group (*p* < 0.05). In addition, both non-silent and silent mutation counts were significantly lower in the SC-high group (*p* < 0.05), indicating a lower overall mutation burden in TNBC rich with SC. In contrast, intratumor heterogeneity was modestly higher in the S -high group (*p* < 0.05).

### SC score is associated with less cell proliferation

**Fig. 4:**
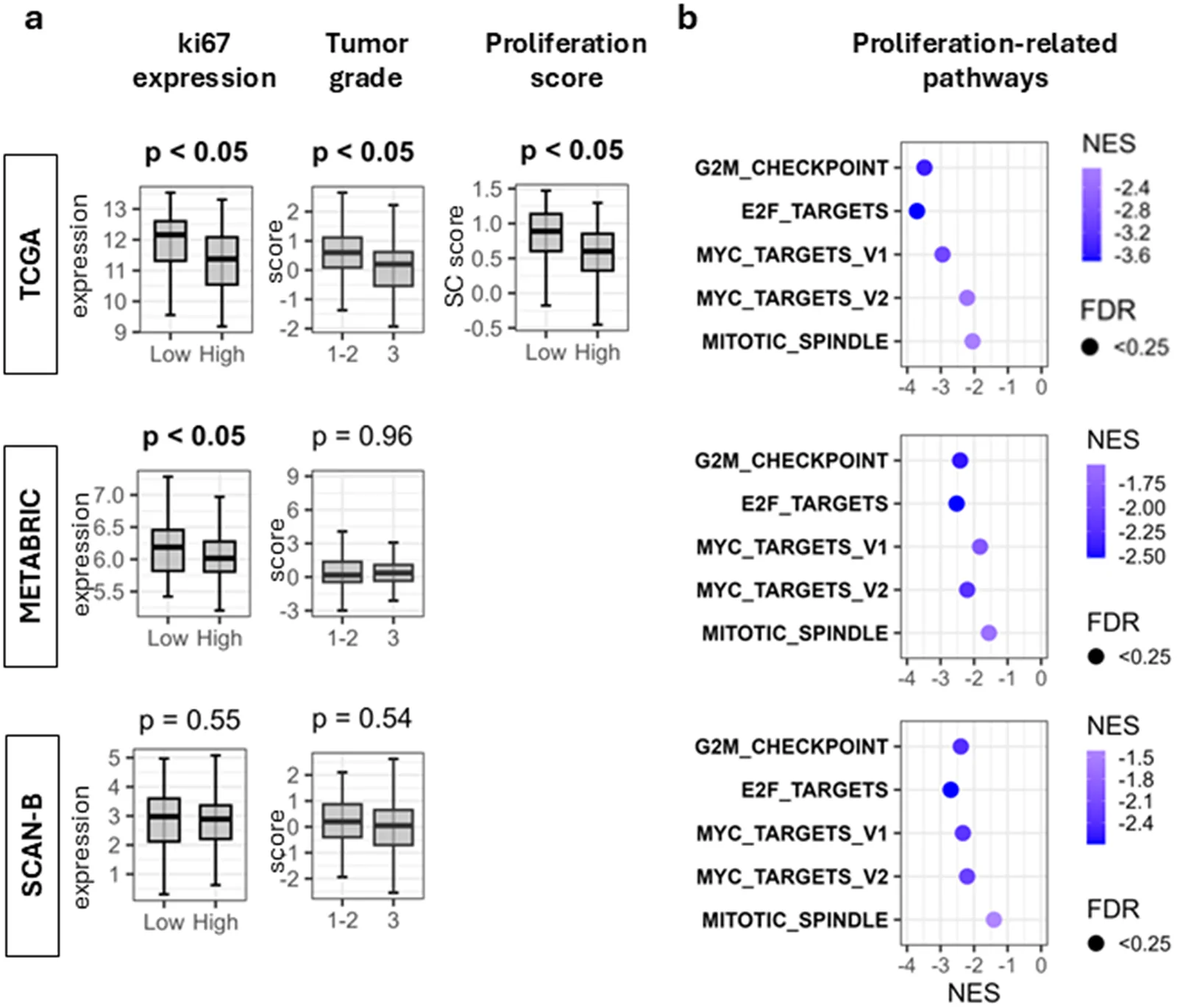
Association of SC score with tumor proliferation across TCGA, METABRIC, and SCAN-B cohorts. (**A**) Box plots showing MKI67 expression between SC score–high and SC score–low tumors (left), SC score distribution according to histological grade (middle), and the proliferation score between SC score–high and SC score–low tumors (right). In the boxplots, the center line indicates the median, the box represents the interquartile range (IQR), and whiskers extend to 1.5×IQR. Outliers were omitted. P values were calculated using the Wilcoxon rank-sum test. (**B**) Dot plots showing normalized enrichment scores (NES) and false discovery rate (FDR) from gene set enrichment analysis (GSEA) for proliferation-related Hallmark gene sets, including G2M checkpoint, E2F targets, MYC targets V1/V2, and mitotic spindle, comparing SC-high and SC-low tumors across the three cohorts. Dot color represents NES and dot size indicates the FDR

Given the previous reports that density of nerves associates with cancer aggressiveness, it was of interest to investigate the relationship between the SC score and cell proliferation in TNBC. Across all three cohorts (TCGA, METABRIC, and SCAN-B), expression of ki67 gene, a marker of cell proliferation, was significantly lower in the SC-high group than in the SC-low group (Fig. 4a; TCGA: *p* < 0.05, METABRIC: *p* < 0.05, SCAN-B: *p* = 0.55). Also, tumors with higher histological grade tended to exhibit lower SC scores, although this association reached statistical significance only in the TCGA cohort (Fig. 4a; TCGA: *p* < 0.05, METABRIC: *p* = 0.96, SCAN-B: *p* = 0.54). In addition, the proliferation score pre-calculated by Thorsson et al., which is based on somatic mutation–associated tumor features, was significantly lower in the SC-high group (Fig. 4a; *p* < 0.05). GSEA further supported these findings. Among the MSigDB Hallmark gene set collection, a curated set of gene signatures representing well-defined biological states, proliferation-related pathways, including G2M check point, E2F targets, MYC target v1/v2, and mitotic spindle, were significantly enriched in the SC-low group (Fig. 4b; all NES < -1.5, all FDR < 0.05). Notably, this inverse association contrasts with prior reports linking nerve density to increased tumor aggressiveness.

### SC score did not demonstrate a consistent association with response to NAC

**Fig. 5:**
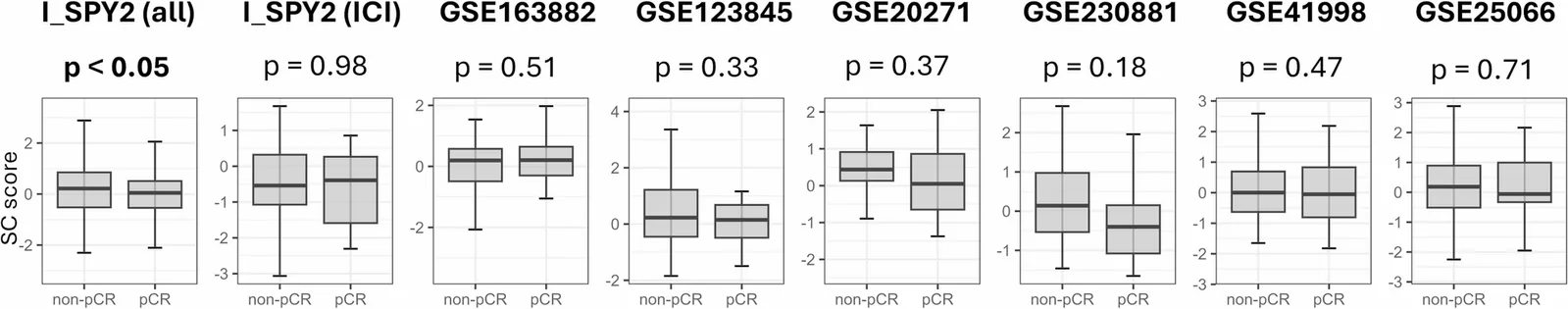
SC scores and therapeutic response status. Box plots comparing SC scores between tumors achieving pathological complete response (pCR) and those with residual disease (non-pCR) across multiple independent TNBC cohorts, including the I-SPY2 trial (all patients and the ICI-treated subset) and publicly available gene expression datasets (GSE163882, GSE123845, GSE20271, GSE230881, GSE41998, and GSE25066). Each box plot shows the distribution of SC scores in pCR and non-pCR tumors within each cohort

Given the lower mutation burden and reduced proliferative activity observed in SC-high tumors, we hypothesized that these tumors may exhibit a poorer response to NAC (Fig. 5). To test this hypothesis, we analyzed seven independent TNBC cohorts treated with anthracycline- and taxane-based NAC, together with the I-SPY2 cohort, which included an immune checkpoint inhibitor (ICI)-containing regimen, and investigated the association between the SC score and pathological complete response (pCR) after NAC (Fig. 5). Overall, the SC score did not show a consistent association with pCR across the analyzed cohorts. Although a significant difference was observed in the overall I-SPY2 cohort, where SC scores were lower in tumors achieving pCR (*p* < 0.05), this association was not observed in the ICI subset or in any of the other independent cohorts. Taken together, these findings suggest that the SC score is not strongly associated with response to NAC in TNBC.

### High SC score is associated with less immune activity, less immune cell infiltrations, and with activated epithelial–mesenchymal transition (EMT)-related pathways

**Fig. 6:**
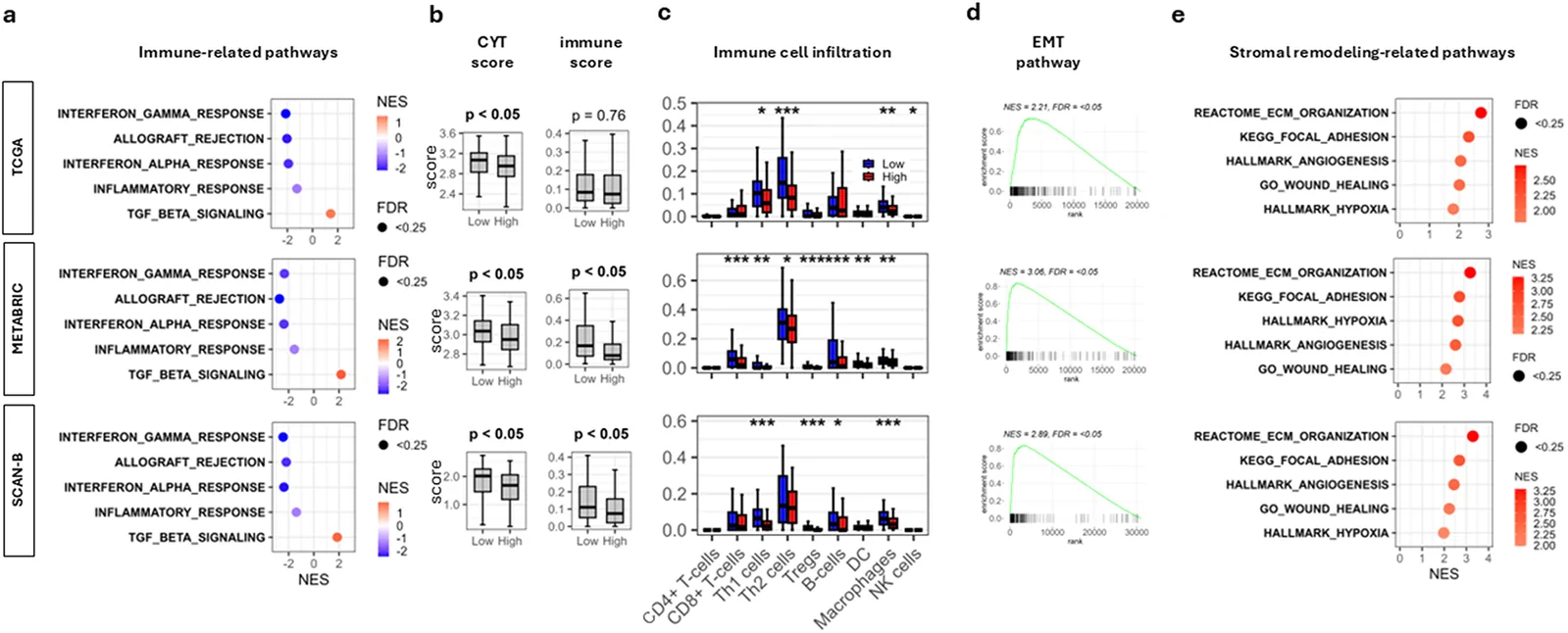
Association of the SC score with immune activity and EMT-related pathway activity. (**A**) Dot plots showing NES and FDR from GSEA for immune-related Hallmark pathways, including interferon gamma response, interferon alpha response, allograft rejection, inflammatory response, and TGF-β signaling, comparing SC score–high and SC score–low tumors across the three cohorts. Dot color represents NES and dot size indicates the FDR. (**B**) Box plots showing differences in cytolytic activity (CYT) score and xCell immune score between SC score–high and SC score–low tumors. In the boxplots, the center line indicates the median, the box represents the interquartile range (IQR), and whiskers extend to 1.5×IQR. Outliers were omitted. P values were calculated using the Wilcoxon rank-sum test. (**C**) Box plots showing infiltration levels of immune cell populations estimated using the xCell algorithm, including CD4⁺ T cells, CD8⁺ T cells, Th1 cells, Th2 cells, regulatory T cells (Tregs), B cells, dendritic cells (DC), macrophages, and natural killer (NK) cells. Red indicates SC-high and blue indicates SC-low tumors. Asterisks denote statistical significance (*p < 0.05, **p < 0.01, *p < 0.001). (**D**) GSEA enrichment plots for the epithelial–mesenchymal transition (EMT) pathway comparing SC -high and SC-low tumors in each cohort. Normalized enrichment score (NES) and false discovery rate (FDR) are shown in each plot. (**E**) Dot plots showing NES and FDR from GSEA for stromal remodeling–related pathways, including extracellular matrix organization, focal adhesion, angiogenesis, wound healing, and hypoxia, across the three cohorts. Dot color represents NES and dot size indicates FDR

Our results that SC high TNBC was associated with lower mutation burden, lower cell proliferation, but not with response to NAC, did not seem consistent with no difference in survival compared with SC low TNBC, since lower cell proliferation with no difference in NAC should result in better survival. This led us to investigate the tumor microenvironmental features that are associated with cancer biology. GSEA revealed that immune-related pathways among Hallmark collection, including interferon alpha response, interferon gamma response, and allograft rejection, were all significantly enriched in the SC-low group (Fig. 6a; all NES <-1.5, FDR < 0.25). In contrast, TGF-beta signaling, a pathway associated with immunosuppression, was significantly enriched in the SC-high group (Fig. 6a; all NES > 1.5, all FDR < 0.25). Consistent with this observation, the cytolytic activity score, a measure of antitumor immune activity, was significantly lower in the SC-high group across all cohorts (Fig. 6b; all *p* < 0.05). Similarly, the xCell immune score tended to be lower in SC-high group, although this difference did not reach statistical significance in the TCGA (Fig. 6b; TCGA *p* = 0.08; METABRIC and SCAN-B both *p* < 0.05).

Analysis of individual immune cell populations using xCell algorithm further demonstrated that the infiltration of several immune cell types, including Th1 cells and macrophages, were significantly lower in the SC-high group across all cohorts (Fig. 6c; all *p* < 0.05). Taken together, these results indicate that SC-high tumors exhibit an immune-cold TME characterized by reduced immune activity and immune cell infiltration.

In line with the reduced immune activity observed in SC-high tumors, we next examined whether the SC score was associated with EMT, a key process linked to stromal remodeling. Across all three cohorts, EMT-related pathways were consistently enriched in the SC-high group (Fig. 6d; all NES > 1.5, FDR < 0.25). Furthermore, pathways related to stromal remodeling, including extracellular matrix organization, focal adhesion, angiogenesis, wound healing, and hypoxia, were also significantly enriched in the SC-high group (Fig. 6e; all NES > 1.5, FDR < 0.25).

## Discussion

This study utilized a publicly available SC signature gene set to generate a reproducible transcriptomic SC score as a surrogate of tumor-associated nerve fibers and investigated its clinical relevance in TNBC. Using spatial transcriptomic data with histologically annotated intratumoral nerves, we confirmed that SC signature genes are spatially restricted to the location where nerve fibers exist, indicating that the SC score reflects the presence of Schwann cells around nerve fibers. Across multiple large transcriptomic cohorts, the SC score showed no significant association with survival outcomes or the frequency of major driver gene mutations. This contrasts with observations in other cancer types, where neural features have been linked to prognosis. Despite the lack of association with survival outcomes, SC-high tumors harbored a lower fraction of genome alteration and reduced mutation burden, consistent with potentially lower tumor immunogenicity. SC-high tumors also consistently exhibited lower proliferative activity; however, this did not translate into a consistent difference in response to neoadjuvant chemotherapy. Additionally, SC-high tumors were characterized by low immune activity and immune cell infiltration, together with enrichment of EMT and stromal remodeling–related pathways. Taken together, these findings suggest that the SC score delineates a distinct TNBC phenotype characterized by neural-associated features, reduced immunogenicity, and stromal remodeling within the TME.

Nerve density within tumors appears to associate with distinct TME phenotypes across multiple cancers, including breast cancer [29, 31], where nerve density has been reported to be higher than in normal mammary tissue [28, 32] and is particularly elevated in TNBC [19], the subtype associated with the poorest clinical outcomes. Experimental studies further suggest that increased nerve density promotes tumor aggressiveness, invasion, and metastasis [19], whereas denervation suppresses tumor growth [33].These effects are mediated by neurotransmitters and neuropeptides released from nerves, thereby altering the TME [1]. In the present study, high SC scores were rather associated with reduced proliferative activity and showed no clear association with survival outcomes. However, these findings should not be interpreted as directly contradicting previous studies. Most previous studies assessed nerve density histologically or used experimental models of advanced or metastatic cancer, whereas the present study evaluated an SC-associated transcriptomic score in human early-stage TNBC. Histological nerve density and the SC score may therefore capture related but distinct aspects of neural biology. Furthermore, although we used SCs as surrogate markers of nerve fibers to quantify neural-associated features transcriptomically, the potential interactions of SCs themselves with tumor and stromal components within the TME should also be considered.

As a major cellular component of peripheral nerves, SCs not only provide structural and functional support to axons but also contribute to the neural microenvironment within tumors [34]. In adult tissues, SCs primarily exist as myelinating or non-myelinating SCs that support and protect peripheral axons [35]. However, following nerve injury, SCs can undergo reversible reprogramming into a repair-like phenotype [36], characterized by increased motility, secretion of trophic factors, and active remodeling of the surrounding microenvironment to support axonal regeneration [37]. Notably, similar phenotypic reprogramming of SCs has also been observed in tumor-associated SCs, where nerves are exposed to tumor-induced injury-like conditions within the TME [35]. In addition to nerve-tumor interactions, SCs themselves also contribute to tumor biology [35, 38]. In multiple cancers, including pancreatic, prostate, and oral cancers, SCs have been reported to promote tumor progression by facilitating EMT, and modulating immune responses within the TME [34, 39]. In this study, consistent with previous reports, SC-high tumors were characterized by enhanced EMT and stromal remodeling programs, as well as suppressed immune activities across multiple cohorts. In particular, pathways related to stromal remodeling, including hypoxia, angiogenesis, and extracellular matrix–related programs, were consistently enriched in SC-high tumors. The SC signature used in this study was curated from genes broadly associated with SC biology and may capture multiple SC phenotypes and activation states, including myelinating, non-myelinating, and repair-like SCs. However, not all genes included in the signature are necessarily specific to SCs, and their expression by other cell populations within the TME may also contribute to the score. Therefore, the SC score should be interpreted as an SC-associated transcriptional signal rather than a direct quantitative measure of SC abundance or nerve density. These findings suggest that SC score may capture SC- and neural-associated signals, together with broader processes linked to mesenchymal transition and immune suppression within the TME of TNBC. One possible explanation for the lack of differences in NAC response or survival, despite the association of the SC score with reduced immune activity, enhanced EMT and stromal remodeling, and lower proliferative activity, is that these opposing biological processes may counterbalance each other. Although the coexistence of these features may appear paradoxical, tumor aggressiveness is not determined solely by proliferative activity. In addition, the clinical context of the analyzed cohorts should be considered. In this study, we primarily analyzed early-stage breast cancers without distant metastasis. Previous studies have shown that intratumoral nerve density increases with cancer progression [40], and that tumor microenvironmental changes, including immune evasion, are further amplified in advanced stage [41]. Therefore, the neural-related features observed in this study may represent an earlier phase of tumor–nerve interaction, in which these biological effects are present at the transcriptomic level but have not yet fully translated into differences in therapeutic response or clinical outcomes. Future validation in advanced or metastatic breast cancer cohorts may therefore be warranted. However, the coexistence of low proliferative activity and reduced immune activity, features often associated with decreased sensitivity to chemotherapy and immunotherapy, together with enhanced EMT and stromal remodeling, may still suggest a biologically distinct phenotype within TNBC with potential for tumor progression and therapeutic resistance.

This study has several limitations. The SC score was derived from canonical SC marker genes and therefore may not fully capture the diversity of tumor-associated SC phenotypes. Although spatial transcriptomic analysis demonstrated that this signature was localized along histologically annotated intratumoral nerve fibers, the SC score represents a transcriptomic inference of SC-associated signals rather than a direct measurement of nerve fibers or SCs themselves. Therefore, whether the SC score accurately correlates with the actual density of intratumoral nerve fibers will require further experimental validation. In addition, because bulk transcriptomic analysis captures gene expression from the entire tumor tissue, it may not accurately resolve cell type–specific features at the individual cell level. While this approach was intended to serve as a surrogate for neural-associated structures within tumors, it is also possible that the SC score partially reflects biological effects arising from SCs themselves, given their known interactions with tumor and stromal components. Therefore, the relative contribution of neural structures versus SC-associated cellular processes cannot be fully distinguished in this study, and further experimental validation will be required.

In conclusion, this study demonstrates that transcriptomic SC signature high TNBC is associated with lower cell proliferation, enhanced stromal remodeling and EMT and an immunosuppressed landscape within tumors. These findings highlight neural-associated tumor microenvironmental features captured through SC score, suggesting a potential role of tumor-nerve interactions in shaping a distinct biology of TNBC. Given the technical challenges in directly assessing intratumoral nerve density by histology, transcriptomic approaches such as the SC score may provide a practical framework for investigating neural-associated tumor biology in future translational studies.

## Materials and methods

### Data acquisition

Clinical, transcriptomic and genomic data from multiple publicly available TNBC cohorts were analyzed. Primary analyses including clinical outcome, immune infiltration profiling, genomic feature characterization, and pathway enrichment analysis were conducted using three large independent breast cancer cohorts: The Cancer Genome Atlas (TCGA, *n* = 170), the Molecular Taxonomy of Breast Cancer International Consortium (METABRIC, *n* = 335) and the Sweden Cancerome Analysis Network- Breast (SCAN-B, *n* = 174). To validate the spatial distribution of SCs, we analyzed a spatial transcriptomics dataset from Zenodo (doi:10.5281/zenodo.8135721) [42]. To investigate the association between the SC score and response to neoadjuvant chemotherapy (NAC), we analyzed seven independent cohorts that underwent NAC: GSE194040 (I-SPY2 trial; *n* = 363) [43], GSE25066 (*n* = 133) [44], GSE163882 (*n* = 90) [45], GSE123845 (*n* = 86) [46], GSE20271 (*n* = 63) [47], GSE230881 (*n* = 49) [48] and GSE41998 (*n* = 149) [49]. TCGA and METABRIC data were downloaded from cBioPortal and UCSC Xena. Data for all other cohorts were obtained from the Gene Expression Omnibus (GEO). As all data used in this study were derived from publicly available, de-identified sources, institutional review board (IRB) approval was exempt.

### Generation of the schwann cell (SC) score

For transcriptomic analyses of the bulk tumor cohorts, the SC score was calculated as the average z-score of a publicly available Schwann cell signature gene set comprising 48 genes obtained from PanglaoDB [50] (Supplementary Table S1), computed for each gene across samples within each cohort. Patients were stratified into SC-high and SC-low groups using the median SC score within each cohort as the cutoff.

### Spatial transcriptomic analysis

For spatial validation, we analyzed a published spatial transcriptomic dataset (Wang et al. [42]). Histopathological annotations provided by the authors were used to identify nerve structures within the tissue section. The spatial distribution of SCs was assessed based on the expression patterns of SC signature genes using the AddModuleScore function in Seurat.

### Cell composition, cytolytic activity score (CYT), and immune-related scores

To estimate the infiltration levels of immune cell populations within the TME, we applied the xCell algorithm [51]. The cytolytic activity (CYT) score was calculated as previously described by Rooney et al. [52] and based on granzyme A (GZMA) and perforin (PRF1) expression. For the assessment of tumor immunogenicity and proliferative activity in the TCGA cohort, we used the precomputed scores reported by Thorsson et al. [53]

### Gene set enrichment analysis (GSEA)

Functional enrichment analysis was performed using gene set enrichment analysis (GSEA) based on differentially expressed genes (DEGs), with the Hallmark gene set collection from the Molecular Signatures Database (MSigDB) [54]. Statistical significance was defined as a false discovery rate (FDR) < 0.25, and enrichment strength was evaluated using the normalized enrichment score (NES).

### Association between sc score and response to neoadjuvant chemotherapy

To evaluate the association between the SC score and response to NAC, we analyzed seven independent TNBC cohorts treated with anthracycline- and taxane-based NAC. Among these cohorts, only the I-SPY2 cohort (GSE194040) included treatment regimens containing immune checkpoint inhibitors (ICI). Pathological complete response (pCR) status was extracted from the original clinical annotations of each cohort. Within each cohort, SC scores were calculated and compared between patients who achieved pCR and those with residual disease (non-pCR).

### Statistical analysis

All statistical analyses and data visualization were performed using R software. Group comparisons were performed using the Kruskal–Wallis test, Mann–Whitney U test, or Fisher’s exact test, as appropriate. Survival analyses were conducted using Kaplan–Meier estimation, and differences between groups were assessed using the log-rank test. A two-sided p-value < 0.05 was considered statistically significant.

## Supplementary Information

Below is the link to the electronic supplementary material.

Supplementary Material 1

## Data Availability

All data analyzed in this study are publicly available. The TCGA breast cancer datasets were obtained from cBioPortal (https://www.cbioportal.org/) and the UCSC Xena platform (https://xenabrowser.net/). Data from the other cohorts were obtained from the GEO database (https://www.ncbi.nlm.nih.gov/geo/) and Zenodo (https://zenodo.org/). No new datasets were generated during this study. The code used for the analyses is available from the corresponding author upon reasonable request.
