# Supplementary material for: Association of tumor Schwann cells with epithelial-mesenchymal transition and immune-suppressive microenvironment in triple-negative breast cancer
Source: Breast Cancer Res Treat. 2026 Aug 10;218(3):37. doi: 10.1007/s10549-026-08050-z (PMC13457385; doi:10.1007/s10549-026-08050-z)
Supplement: Supplementary file 1 [file 10549_2026_8050_MOESM1_ESM.pdf]

Supplementary Table S1

gene symbol	gene name
MPZ	Myelin protein zero
ITGB4	Integrin subunit beta 4
LAMC1	Laminin subunit gamma 1
S100B	S100 calcium binding protein B
POU3F1	POU class 3 homeobox 1
GFAP	Glial fibrillary acidic protein
MAG	Myelin associated glycoprotein
OLIG1	Oligodendrocyte transcription factor 1
TST	Thiosulfate sulfurtransferase
NDRG1	N-myc downstream regulated 1
CTHRC1	Collagen triple helix repeat containing 1
NF2	Neurofibromin 2
SEPT9	Septin 9
AGR2	Anterior gradient 2
NGFR	Nerve growth factor receptor
CLDN19	Claudin 19
PRX	Periaxin
NTRK1	Neurotrophic receptor tyrosine kinase 1
ART3	ADP-ribosyltransferase 3
BCAS1	Breast carcinoma amplified sequence 1
NPY	Neuropeptide Y
ASPA	Aspartoacylase
LGI4	Leucine rich glioma inactivated 4
BAG3	BCL2 associated athanogene 3
RXRG	Retinoid X receptor gamma
SOX10	SRY-box transcription factor 10
CMTM5	CKLF like MARVEL transmembrane domain containing 5
BCHE	Butyrylcholinesterase
EGFL8	EGF like domain multiple 8
GFRA3	GDNF family receptor alpha 3
SEMA3B	Semaphorin 3B
MATN2	Matrilin 2
SCN7A	Sodium voltage-gated channel alpha subunit 7
CD82	CD82 molecule
TAGLN	Transgelin
NOV	Nephroblastoma overexpressed
ALDOC	Aldolase, fructose-bisphosphate C
APOD	Apolipoprotein D
CRYAB	Crystallin alpha B
PLP1	Proteolipid protein 1
PMP22	Peripheral myelin protein 22
CADM4	Cell adhesion molecule 4
KCNA1	Potassium voltage-gated channel subfamily A member 1
CNP	2',3'-cyclic nucleotide 3' phosphodiesterase
GPR37L1	G protein-coupled receptor 37 like 1
NRN1	Neuritin 1
CD59A	CD59 molecule
STMN1	Stathmin 1

Supplementary Table S1. List of genes comprising the Schwann cell (SC) signature used for SC score calculation.
